# Umwelt und Gesundheit – Voraussetzungen für Sicherheit und Resilienz. Positionspapier der Kommission Environmental Public Health

**DOI:** 10.1007/s00103-026-04303-w

**Published:** 2026-09-24

**Authors:** Annette Peters, Hajo Zeeb, Caroline Herr, Wolfgang Straff, Claudia Traidl-Hoffmann, Tobia Lakes, Claudia Hornberg, Geschäftsstelle der Kommission Environmental Public Health, Gabriele Bolte, Caroline Herr, Astrid Heutelbeck, Henk Hilderink, Barbara Hoffmann, Claudia Hornberg, Thomas Kraus, Tobia Lakes, Andreas Matzarakis, Odile Mekel, Dennis Nowak, Annette Peters, Martina Ragettli, Doreen Reifegerste, Alexandra Schneider, Wolfgang Straff, Claudia Traidl-Hoffmann, Hajo Zeeb

**Affiliations:** 1 Institut für Epidemiologie, Helmholtz Zentrum München, München, Deutschland; 2 Leibniz-Institut für Präventionsforschung und Epidemiologie – BIPS und Universität Bremen, Bremen, Deutschland; 3 Landesamt für Gesundheit und Lebensmittelsicherheit Bayern, München, Deutschland; 4 Fachgebiet Umweltmedizin und gesundheitliche Bewertung, Umweltbundesamt (UBA), Dessau-Roßlau, Deutschland; 5 Medizinische Fakultät – Institut für Umweltmedizin und Integrative Gesundheit, Universitätsklinikum Augsburg, Augsburg, Deutschland; 6 Geographisches Institut, Humboldt-Universität zu Berlin, Berlin, Deutschland; 7 Medizinische Fakultät OWL, Universität Bielefeld, Bielefeld, Deutschland; 8 Robert Koch-Institut, Gerichtstr. 27, 13347 Berlin, Deutschland

**Keywords:** Öffentliche Gesundheit, Krisen, Ökosystem, Klimawandel, Biodiversität, Public health, Crises, Ecosystem, Climate change, Biodiversity

## Abstract

Negative Einflüsse aus der Umwelt auf die menschliche Gesundheit, die in den Gesundheitswissenschaften im Fokus stehen, sind vor allem die Folge menschlicher Eingriffe in Ökosysteme. Dies wird in globalen und lokalen Problemen wie Klimawandel, Umweltverschmutzung, Biodiversitätsverlust, auftretenden Pandemien oder den zahlreichen gesundheitlichen Folgen von Kriegen sichtbar. In Zeiten multipler Krisen sollte das Zusammenspiel von Umwelt und Gesundheit im Fokus stehen, da es Grundlage für eine resiliente Gesellschaft ist.

In einer politisch wie auch ökologisch angespannten globalisierten Welt wird deutlich, dass Umwelt‑, Gesundheits- und Sicherheitsfragen eng miteinander verflochten sind. Eine intakte Umwelt stellt nicht nur ein ökologisches und ethisches Ziel dar, sondern fungiert als zentrale Gesundheitsressource, die wesentlich zur Stabilität und Sicherheit moderner Gesellschaften beiträgt.

Anhand dreier beispielhafter Bereiche von Klimawandel und Gesundheit, Luftschadstoffe und Gesundheit sowie Biodiversität und Gesundheit werden Schlussfolgerungen für umwelt- und gesundheitsbezogene Sicherheitsfragen aufgezeigt. Mit Bezug auf diese Erkenntnisse trifft die Kommission Environmental Public Health zusammenfassend konkrete Empfehlungen zur Sicherung und Fortführung der sektorenübergreifenden Zusammenarbeit zu Umwelt und Gesundheit.

## Abstract (en)

Negative environmental impacts on human health—which are a central focus of the health sciences—are primarily the result of human interference with ecosystems. This is evident in global and local issues such as climate change, environmental pollution, biodiversity loss, emerging pandemics, and the numerous health consequences of war. In times of multiple crises, the interplay between the environment and health should be a priority, as it forms the foundation for a resilient society.

In a globalized world marked by political and ecological tensions, it is clear that environmental, health, and security issues are closely intertwined. An intact environment is not only an ecological and ethical goal but also serves as a central health resource that contributes significantly to the stability and security of modern societies.

Using three illustrative areas—climate change and health, air pollutants and health, and biodiversity and health—conclusions regarding environmental and health-related security issues are presented. Based on these findings, the Environmental Public Health Commission summarizes specific recommendations for ensuring and continuing cross-sectoral cooperation on environment and health.

## Hintergrund

In Zeiten multipler Krisen sollte das Zusammenspiel von Umwelt und Gesundheit im Fokus stehen, da es eine zentrale Voraussetzung für die Resilienz, Sicherheit und Zukunftsfähigkeit Deutschlands ist. Als hochindustrialisierter und zugleich von den Folgen des Klimawandels, demografischen Veränderungen, globalen Gesundheitsrisiken und geopolitischen Spannungen betroffener Staat ist Deutschland in besonderem Maße auf stabile Umwelt- und Gesundheitsbedingungen angewiesen.

Die Notwendigkeit, mit multiplen Krisen umzugehen, stellt unsere Gesellschaft aktuell vor große Herausforderungen. Umso mehr gilt es, die Anfälligkeit der gesellschaftlichen Teilsysteme gegenüber externen Schocks zu reduzieren. Die Verwundbarkeit des Gesundheitssektors wurde bereits während der Corona-Pandemie deutlich. Sie wird aber angesichts der Risiken, die durch die ökologische Krise entstehen, ungleich höher werden. Um die Funktionsfähigkeit des Gesundheitssystems zu bewahren, sollte die Stabilität des Ökosystems daher als Teil einer integrierten Sicherheitsstrategie verstanden werden. Aus Sicht der Kommission ist es entscheidend, aktuelle und künftige Risikofaktoren in diesem Kontext zu erkennen und vorausschauend zu handeln. Im Interesse einer resilienten Gesellschaft bedeutet das für die Gesundheitspolitik, Umwelt- und Gesundheitsaspekte in allen Politikbereichen zu berücksichtigen. Daher ist die Einbeziehung von Umwelt- und Gesundheitsaspekten in alle Politikbereiche („Health in all Policies“ – HiAP) insbesondere bei komplexen Krisen und auch bezogen auf Sicherheitsstrategien bedeutsam.

Wenn Umwelt- und Gesundheitsprobleme in Krisen nicht ausreichend beachtet werden, können sie sich gegenseitig verstärken und die politische Stabilität im In- und Ausland gefährden. Aus Sicht der Kommission ist es daher insbesondere angesichts der aktuellen schwierigen Rahmenbedingungen entscheidend, zentrale Aspekte des Zusammenspiels von Gesundheit und Umwelt und ihre besondere Bedeutung für die Vorsorge, Krisenfestigkeit und nachhaltige Entwicklung Deutschlands hervorzuheben.

Unsere Gesundheit wird beeinflusst von den Umweltbedingungen, die uns umgeben und in Verbindung mit unserem individuellen Gesundheitsverhalten stehen. Für die Kommission umfasst der Begriff „Umwelt“ mehr als nur die natürlichen Ökosysteme. Verschiedene Umweltbereiche können sich in vielfältiger Weise direkt oder indirekt (über soziokulturelle Faktoren vermittelt) auf die menschliche Gesundheit auswirken. Die Umwelt kann Gesundheit aktiv fördern, zum Beispiel durch die Erbringung von Ökosystemdienstleistungen wie die Bereitstellung von Nahrung und sauberem Trinkwasser oder in Form von Orten, die der körperlichen Aktivität dienen. Auch können gesundheitsschädigende Einflüsse abgemildert oder verhindert werden.

Negative Einflüsse aus der Umwelt auf die menschliche Gesundheit, die in den Gesundheitswissenschaften im Fokus stehen, sind vor allem die Folge menschlicher Eingriffe in Ökosysteme. Dies wird in globalen und lokalen Problemen wie Klimawandel, Umweltverschmutzung, Biodiversitätsverlust, auftretenden Pandemien oder den zahlreichen gesundheitlichen Folgen von Kriegen sichtbar. Die unterschiedliche Exposition verschiedener Bevölkerungsgruppen hinsichtlich umweltbedingter Risiken und ihr Zugang zu gesundheitsförderlichen Umweltbedingungen spielen bei der Betrachtung der Zusammenhänge zwischen Umwelt und Gesundheit eine zentrale Rolle und finden sich im Querschnittsbereich der Umweltgerechtigkeit wieder.

Wenn präventive Maßnahmen über alle Bereiche hinweg nachhaltig umgesetzt werden, entstehen gesunde und gerechte Lebensbedingungen. Auch aus einer gesundheitsökonomischen Perspektive ist das relevant, denn Vernachlässigung von Umwelt- und Gesundheitsschutz verursacht langfristig hohe Kosten und mehr Krankheits- sowie Ausfalltage.

In einer politisch wie auch ökologisch angespannten globalisierten Welt wird deutlich, dass Umwelt‑, Gesundheits- und Sicherheitsfragen eng miteinander verflochten sind. Eine intakte Umwelt stellt nicht nur ein ökologisches und ethisches Ziel dar, sondern fungiert als zentrale Gesundheitsressource, die wesentlich zur Stabilität und Sicherheit aller Gesellschaften beiträgt. Sie bildet eine zentrale Voraussetzung für den Erhalt der menschlichen Gesundheit und das Wohlergehen einer Bevölkerung [1, 2]. Der Rückgang der Biodiversität und funktionaler Lebensräume, die vielfältigen Umweltbelastungen durch gesundheitsschädliche Stoffe in Wasser, Boden und Luft sowie die Folgen des Klimawandels ergeben eine gesundheitspolitische Herausforderung, welche auch dann besteht, wenn andere Themen zwischenzeitlich dringlicher erscheinen. Mittel- und langfristig werden diese Krisen nur durch geschickte politische Entscheidungen auf der Basis intensiver Forschung und Entwicklung lösbar sein. Umwelt und Gesundheit zusammenzudenken ist gerade unter aktuellen politischen Entwicklungen entscheidend in Deutschland, da sie wichtige Ressourcen für die innere Sicherheit, z. B. im Fall von Katastrophen, und äußere Sicherheit, z. B. im Fall von geopolitischen Bedrohungen, darstellen.

## Beispiele für Zusammenhänge zwischen Umwelt, Gesundheit und Sicherheit

Trotz Erfolgen im Umweltschutz bestehen in Deutschland weiterhin unmittelbare Gesundheitsrisiken aufgrund von negativen Umwelteinflüssen für die Bevölkerung [3]. Dazu gehören Luft- und Wasserverschmutzung (z. B. durch Mikroplastik, Chemikalien, Arzneimittel, Dünger und Pestizide in Gewässern) sowie Lärmbelastung, Hitze- und Belastungen durch Ultraviolettstrahlung (UV-Belastungen), Flutereignisse, sich ausweitende Antibiotikaresistenzen und ein zunehmender Verlust der Biodiversität. Auch der oft fehlende oder erschwerte Zugang zu Umweltressourcen wie öffentliche Grünflächen bleibt gerade für vulnerable Gruppen ein substanzielles Problem. Studien zeigen, dass Umweltverschmutzung international jährlich Millionen vorzeitige Todesfälle verursacht und Umweltbelastungen auch zu signifikant erhöhter Morbidität und verringertem Wohlbefinden beitragen [4]. Der Klimawandel verschärft diese Belastungen durch Hitzestress, erhöhte UV-Strahlung, begünstigte Ausbreitung von Infektionskrankheiten, Ernährungsunsicherheit und psychische Erkrankungen [5–7]. Diese Erkenntnisse sind angesichts der zunehmenden Alterung der Gesellschaft von besonderer Relevanz.

In einigen Bereichen sind die gesundheitsgefährdenden Auswirkungen negativer Umwelteinflüsse seit Langem bekannt und es sind bereits Maßnahmen zum verbesserten Umweltschutz sowie zur Prävention umweltbedingter Erkrankungen eingeleitet worden. So wurden insbesondere in der Luftreinhaltung deutliche Erfolge erzielt. Trotzdem ist beispielsweise Feinstaub immer noch einer der gesundheitsrelevantesten Luftschadstoffe. Die auf breiter wissenschaftlicher Basis erarbeiteten Richtwerte der Weltgesundheitsorganisation (WHO; [8]) werden in zahlreichen deutschen Städten weiterhin deutlich überschritten (siehe [9]). Feinstaub wurde im Rahmen des Global-Burden-of-Disease-(GBD-)Projektes im Mai 2024 als wichtigster Risikofaktor für die Gesamtkrankheitslast weltweit identifiziert [10]. In anderen Handlungsfeldern fehlen wirksame Maßnahmen noch oder sind nur unzureichend umgesetzt, wie z. B. im Klima- und Biodiversitätsschutz.

Die Bedeutung von Umwelt und Gesundheit wird im Vergleich zu anderen Themen im Kontext von innerer und äußerer Sicherheit oft unterschätzt. Dies zeigt sich derzeit weltweit in politischen Planungen und Programmen, welche einen Fokus im Bereich der Sicherheit sehen, jedoch die Umwelt und die Verflechtungen mit der Gesundheit als Sicherheitsressource zum Teil nicht berücksichtigen. Dazu zählen Klimapolitikabschwächungen der Europäischen Union (EU), wie beispielsweise die Aufweichung des Verbots von per- und polyfluorierten Chemikalien (PEFAS), als eine Priorisierung wirtschaftlicher Sicherheit vor langfristiger Umwelt- und Gesundheitssicherheit. Eine Rolle spielt auch der Abbau von Umweltstandards in Krisenzeiten: Mit Argumenten zu Sicherheitsaspekten wird in Zeiten hoher Energiepreise oder geopolitischer Spannungen der Umweltschutz oft kurzfristig zurückgefahren (z. B. Rückkehr zu Kohle- oder Gaskraftwerken, Lockerung von Naturschutzauflagen und Umweltstandards sowie beim Infrastruktur-Zukunftsgesetz, welches sich auf die Gewährleistung der öffentlichen Sicherheit beruft). Dabei werden die gesundheitlichen Langzeitfolgen in die Sicherheitsbilanz nicht einbezogen. Dies betrifft ebenso den Wiederaufbau nach Konflikten und Kriegen.

Soziale, wirtschaftliche und gesundheitliche Risiken können die innere Sicherheit eines Staates gefährden. So können Umweltzerstörung und ungleiche Umweltbelastungen soziale Spannungen intensivieren und Vertrauen in staatliche Institutionen schwächen [11]. Dagegen trägt eine intakte, gesundheitsförderliche Umwelt zu sozialem Frieden bei, da sie die Lebensqualität erhöht, über umweltbezogene Prävention Gesundheitskosten senkt und psychische Belastungen reduziert.

Auch im Bereich der äußeren Sicherheit nehmen Umwelt- und Klimaschutz eine wichtige Rolle ein. Der Sicherheitsrat der Vereinten Nationen (UN-Sicherheitsrat) erklärte bereits 2007, dass der Klimawandel ein „Bedrohungsmultiplikator“ für internationale Stabilität ist [12]. Konflikte um Wasser, Nahrungsmittel und Land werden durch ökologische Krisen verschärft und können zu Migration, Verlust staatlicher Autorität und Strukturen sowie letztlich auch zu militärischen Auseinandersetzungen führen. Die NATO (North Atlantic Treaty Organization „Organisation des Nordatlantikvertrags“) bezeichnet in ihrem strategischen Konzept [13] den Klimawandel sogar als die größte Herausforderung für die Sicherheit der NATO-Mitgliedstaaten. Der Klimawandel wirkt sich direkt auf die militärische Infrastruktur und Ausrüstung sowie indirekt auf Missionsprofile, militärische Lieferketten und das Sicherheitsumfeld des Bündnisses aus [13].

Der Wissenschaftliche Beirat der Bundesregierung Globale Umweltveränderungen (WBGU) äußert sich in seinem aktuellen Politikpapier dazu: „Nur ein integriertes Sicherheitskonzept, das neben dem unmittelbaren Schutz vor Krieg und Gewalt auch den weltweiten Schutz natürlicher Lebensgrundlagen wie Klima und Biodiversität sowie globale Gerechtigkeit, gesellschaftlichen Zusammenhalt und Frieden umfasst, kann langfristig erfolgreich sein“ [14]. Bereits in der Ottawa-Charta 1986 wurde neben einem stabilen Ökosystem auch Frieden als Gesundheitsdeterminante benannt. Einen Beitrag dazu können auch gemeinsame Umweltinitiativen leisten, die durch ihre Arbeit Friedensprozesse und internationale Kooperation fördern [15].

Damit wird deutlich, dass Maßnahmen zum Schutz von Umwelt und Gesundheit sowie die gemeinsame Betrachtung von Umwelt- und Gesundheitsthemen Staaten sowohl im Inneren als auch nach außen stabilisieren, indem Konfliktursachen gemindert und Resilienz gegenüber lokalen und globalen Risiken gestärkt wird. Ein langfristiges, kontinuierliches, effizientes und an die jeweiligen gesundheitlichen Problemlagen angepasstes Handeln ist dabei notwendig.

## Schlussfolgerungen für umwelt- und gesundheitsbezogene Sicherheitsfragen

### 1. Klimawandel und Gesundheit

Das Ausmaß und die Vielfalt an gesundheitlichen Auswirkungen im Zusammenhang mit den Folgen des globalen Klimawandels sind mittlerweile unbestritten. Im aktuellen Lancet Countdown Report 2025 [16] und im Sachstandsbericht „Klimawandel und Gesundheit 2023“ des Robert Koch-Instituts (RKI; [7]) wird auf die unterschiedlichen Risiken wie die weiter zunehmende Anzahl an Tagen mit hohen Lufttemperaturen und erhöhter UV-Strahlung ebenso wie auf das verstärkte Auftreten von durch Mücken übertragenen Erkrankungen hingewiesen. Das Gleiche gilt für die zunehmende Bedrohung der Landwirtschaft durch Dürren oder Überflutungen und die damit einhergehenden Folgen für die Versorgung mit Lebensmitteln. Das Umweltbundesamt (UBA) weist zudem auf die zunehmende Hitzebelastung vor allem in Städten und die davon besonders betroffenen vulnerablen bzw. besonders exponierten Gruppen hin, ebenso wie auf zunehmende Allergien und die Gefahr durch Mikroorganismen und Algen [17]. Die Problematik der sozioökonomischen Ungleichheiten in der Exposition gegenüber Klimawandelfolgen, in der Empfindlichkeit gegenüber den gesundheitlichen Wirkungen und in den Anpassungskapazitäten spielt global wie regional eine wichtige Rolle [1, 18–20]. Auch Zusammenhänge zwischen der Klimakrise und der psychischen Gesundheit gewinnen an Bedeutung [21].

Allerdings gibt es in den Bereichen Klimaschutz und Klimaanpassung auch viele Chancen und erste Erfolge. Die Chancen einer „ökosaluten“ Politik, die Synergien von Naturschutz und Gesundheit stärkt und umsetzt, wurden schon vom Sachverständigenrat für Umweltfragen (SRU) skizziert und die Aufmerksamkeit für die gesundheitlichen Folgen der Klimakrise nimmt zu. Immer mehr Städte und Gemeinden stellen ihre Hitzeaktionspläne fertig [1] und berücksichtigen dabei gesundheitliche Chancengerechtigkeit (z. B. Hitzeaktionsplan Bremen und Bremerhaven im Rahmen der Klimaanpassungsstrategie Bremen [22]). Hitzeschutz- und andere Anpassungsmaßnahmen an die Folgen des Klimawandels durch die Schaffung, Erhaltung und Pflege grüner Räume in Städten haben auch zusätzliche positive Effekte auf das Sicherheitsgefühl der Bevölkerung sowie auf die Anzahl von Kriminalfällen [23].

### 2. Luftschadstoffe und Gesundheit

Die Luftqualität und ihre Auswirkungen auf die Gesundheit sind ein seit Jahrzehnten intensiv beforschtes und diskutiertes Feld [24]. Die WHO hat in den Air Quality Guidelines (2021) ihre Empfehlungen für die Richtwerte zum Schutz der Gesundheit vor Feinstaub‑, Stickstoffdioxid- und Ozon-Expositionen gegenüber früheren Empfehlungen deutlich abgesenkt [8]. Dies erfolgte auf Basis der Ergebnisse von weltweiten Studien, die einen linearen Zusammenhang zwischen Luftschadstoffkonzentrationen und der Sterblichkeit robust belegen. Demnach besitzen bereits Konzentrationen eine gesundheitliche Relevanz, die deutlich unterhalb der Werte liegen, die heute noch regelmäßig in zahlreichen deutschen Städten gemessen werden [8]. Dabei spielen auch Umweltgerechtigkeitsaspekte eine Rolle, da unterschiedliche Expositionen gegenüber Luftschadstoffen innerhalb von Städten existieren.

Auch wenn die Luftreinhaltung insgesamt eine Erfolgsgeschichte dafür ist, wie nationale und internationale Forschung sowie nationale politische und gesellschaftliche Akteure den Schutz der Bevölkerung vor Umweltschadstoffen vorantreiben können, bleibt insbesondere im urbanen Raum und mit Blick auf Fragen der sozialen Gerechtigkeit noch einiges zu tun. So sind besonders sozioökonomisch schlechter gestellte Bevölkerungsgruppen darauf angewiesen, in städtischen Regionen zu wohnen, die wesentlich stärker vom Auto- und Transportverkehr belastet sind als andere. Insofern kommen hier nachhaltige Mobilitätskonzepte zukünftig stärker zum Tragen zum Erreichen gesundheitlicher Chancengleichheit [25].

In Wohngebieten, die durch den Betrieb von Einzelraumfeuerungsanlagen geprägt sind, kommt es gerade in den Übergangsmonaten zu bestimmten Tageszeiten zu hohen Spitzenkonzentrationen von Feinstäuben durch die Verbrennung von Holz. Nach Angaben des UBA gab es in Deutschland 2023 allein 11,7 Mio. solcher Einzelraumfeuerungsanlagen. Insbesondere ältere und schlecht betriebene Holzöfen verursachen um ein Vielfaches höhere Emissionen als Gas- oder Ölheizungen. Wie viel Feinstaub tatsächlich ausgestoßen wird, hängt dabei nicht nur von Art und Alter der Anlage ab. Auch die Art der Befeuerung, der Wartungszustand der Anlagen sowie die Auswahl und Qualität des genutzten Holzes spielen eine Rolle. Gerade in reinen Wohngebieten kommt es so sehr häufig zu starken Belastungen der lokalen Bevölkerung, die zumeist nicht von der Luftqualitätsüberwachung adäquat erfasst werden.

Durch die Folgen des Klimawandels können zudem bestimmte Schadstoffe in der Zukunft relevanter werden: Für Feinstäube gilt dies aufgrund des zu erwartenden häufigeren Auftretens von Waldbränden, welche zu einer sehr hohen und großflächigen Belastung mit Feinstäuben und anderen Verbrennungsprodukten führen können. Außerdem kann durch eine vermehrte Sonneneinstrahlung die Entstehung von bodennahem Ozon begünstigt werden.

Aufgrund erhöhter CO_2_-Konzentration produzieren viele Pflanzen mehr und größere Pollen. Zugleich steigern die CO_2_-Konzentration und Schadstoffe wie Ozon und Feinstaub in der Luft das Allergiepotenzial der Pollen [26]. Darüber hinaus verändern sich durch den Klimawandel die Zeiten des Pollenflugs, die Pollenkonzentrationen, das Pollenspektrum, die Allergenität der Pollen sowie die Ansiedelung bisher nicht heimischer Pflanzenarten [7].

Zudem können durch Klimawandelfolgen vermehrt Saharastäube Deutschland erreichen und zu erhöhten Feinstaubbelastungen führen. Aber auch durch Extremwetterereignisse in Deutschland selbst können aufgrund von Desertifikationsprozessen, welche durch Starkregenereignisse, aber auch durch Dürren begünstigt werden, enorme Staubaufwirbelungen entstehen, welche die Gesundheit belasten.

Aktuelle Ergebnisse belegen die Verstärkung von Hitzeeffekten durch erhöhte Luftschadstoffkonzentrationen und ihre Auswirkungen auf die Gesundheit [27]. Aus diesen Gründen empfiehlt die Kommission dem RKI und dem UBA, dieses Handlungsfeld weiter prioritär zu betrachten und die Synergien mit anderen Handlungsfeldern sichtbar zu machen. Hier bestehen z. B. große Potenziale im Zusammenhang mit der Erstellung und Umsetzung nachhaltiger Mobilitätskonzepte im urbanen Raum, aber auch mit der Aufklärung der Bevölkerung im Hinblick auf die Vermeidung unnötiger Luftbelastungen. Beispielsweise werden Silvesterfeuerwerk, Lagerfeuer, Holzofennutzung sowie der Betrieb von benzinbetriebenen Gartengeräten oder Laubbläsern in der Bevölkerung hinsichtlich der damit verbundenen Gesundheitsrisiken kaum wahrgenommen.

Die Kommission Environmental Public Health empfiehlt aus diesen Gründen die dringende Umsetzung der empfohlenen WHO-Richtwerte [28].

Letztlich ist auch zu bedenken, was Krisen und Kriege für die Luftbelastung zusätzlich bedeuten. In Kriegen kommt es üblicherweise zu massiven Luftbelastungen, ausgelöst z. B. durch Explosionen, Brände, zerstörte Industrieanlagen, Treibstofflager und militärische Logistik. Ausgedehnte Waldbrände wie auch Zerstörungen großer Industrieanlagen haben Emissionen zur Folge, die Ländergrenzen überschreiten. Die ausgedehnten Waldbrände in Kanada in den Jahren 2023 und 2025 führten zu einer ausgeprägten zusätzlichen Feinstaubbelastung sowohl in den USA wie auch in Europa [29]. Längerfristig bestehende Luftbelastungen schwächen die Gesundheit ganzer Bevölkerungen – was die gesellschaftliche und auch staatliche Resilienz reduziert. Luftbelastungen für sich genommen, aber besonders in Kombination mit Hitzewellen und anderen Extremwettereignissen senken so die Fähigkeit eines Landes, auf Krisen oder Konflikte zu reagieren.

### 3. Biodiversität und Gesundheit

Biodiversität wird im Übereinkommen über die biologische Vielfalt der Vereinten Nationen von 1992 als Vielfalt lebender Organismen aus verschiedener Herkunft wie Land‑, Meeres- und sonstigen aquatischen Ökosystemen definiert. Dies schließt die Vielfalt innerhalb und zwischen Arten sowie die Diversität der Ökosysteme ein. Die bis heute geltende Definition von Gesundheit, die 1948 von der WHO formuliert wurde, beschreibt Gesundheit als einen Zustand vollständigen körperlichen, geistigen und sozialen Wohlbefindens, der über die Abwesenheit von Krankheit hinausgeht [30]. Biodiversität und Gesundheit stehen in einem engen Zusammenhang.

Das Intergovernmental Science-Policy Platform on Biodiversity and Ecosystem Services (IPBES) Globale Assessment von 2019 warnt vor einem beispiellosen Artensterben: Etwa eine Million Tier- und Pflanzenarten sind dem Bericht zufolge innerhalb von Jahrzehnten vom Aussterben bedroht, wenn nicht Maßnahmen zur Reduzierung der treibenden Faktoren des Biodiversitätsverlusts wirksam werden. Der Bericht identifiziert Landnutzungsänderungen, direkte Ausbeutung, Klimawandel, Umweltverschmutzung und invasive Arten als Hauptursachen und fordert einen transformativen Wandel unserer Systeme [31].

Eine biodiverse Umwelt kann die Gesundheit von Menschen auf unterschiedliche Arten und Weisen schützen oder fördern: Eine biodiverse Vegetation mit hoher struktureller Komplexität und Dichte stelle eine wirksame Barriere gegen ultrafeine Partikel aus Verbrennungsprozessen dar [32]. Zudem gibt es zunehmend Hinweise darauf, dass Biodiversität einen schützenden Einfluss auf die Anfälligkeit gegenüber Infektionskrankheiten hat. Das gilt insbesondere bei Erkrankungen, die direkt oder indirekt zwischen Tier und Mensch übertragen werden können (vektorübertragene Zoonosen) und damit einhergehenden Epidemien [33]. Dies ist u. a. auf den sogenannten Verdünnungseffekt zurückzuführen [34]: In artenreichen Gebieten gibt es – auf die gesamte Artenvielfalt bezogen – mehr Wirte, welche Krankheitserreger *nicht* übertragen. Dadurch sinkt die Wahrscheinlichkeit, dass Vektoren infizierte Wirtsorganismen finden. Dies führt wiederum zu einer Reduzierung der Infektionsrate in der Vektorpopulation und somit zu einem geringeren Infektionsrisiko für die Menschen.

Ein wichtiger gesundheitserhaltender Faktor ist auch die Besiedlung der Haut, des Darms und anderer Organe des Menschen mit förderlichen Mikroorganismen: Die Biodiversitätshypothese legt nahe, dass symbiotische Mikroorganismen im menschlichen Mikrobiom zu einer gesunden Entwicklung des Immun- und Verdauungssystems beitragen können [35]. Die Diversität des menschlichen Mikrobioms wird dabei von der genetischen Disposition, der Umwelt des Einzelnen sowie seinem Lebensstil, insbesondere der Ernährung, beeinflusst. Menschen, die in städtischen Gebieten leben, haben im Vergleich zu ländlich lebenden Menschen weniger Möglichkeiten, über die Haut, die Nahrung oder auch über die Atemwege mit nützlichen Mikroorganismen in Kontakt zu treten [35].

Das Bevölkerungswachstum und die Urbanisierung stören die Ökosysteme und reduzieren oftmals deren Biodiversität. Diese Veränderungen führen mit hoher Wahrscheinlichkeit zu einer Verarmung des menschlichen Mikrobioms [36] mit negativen Auswirkungen auf die Immunregulation und die Homöostase [36], was sich bspw. in der Zunahme von Allergien und chronischen Entzündungskrankheiten zeigt. Dies unterstreicht erneut die Notwendigkeit, präventive Strategien zu entwickeln.

Wichtig ist es zu betonen, dass Biodiversität nicht mit grüner und blauer Infrastruktur gleichzusetzen ist [37], wenngleich diese im urbanen Bereich biodiversitätsfördernd sein können. Blaue und grüne Infrastrukturen sind oftmals strukturreich gestaltete Räume. Grünflächen und ein lokales ökologisches Regenwassermanagement, mit welchem Siedlungsflächen widerstandsfähiger gegen die Folgen des Klimawandels werden sollen, können die Biodiversität eines Habitats fördern. Die naturnahe Gestaltung von blau-grüner Infrastruktur ist nicht nur eine aktive Klimaanpassung und Biodiversitätsförderung, sondern stärkt gleichzeitig die Schaffung von Erholungs- und Freizeiträumen [38]. So werden die Lebens- und Aufenthaltsqualität vor Ort gesteigert, was zur Stressreduktion beiträgt, das Risiko von kardiovaskulären Erkrankungen senken kann und sowohl körperliche Aktivität als auch soziale Kohäsion fördert [39, 40].

Der Verlust von Biodiversität ist Teil der planetaren Dreifachkrise, die auch den Klimawandel und die Emissionen neuartiger Chemikalien umfasst [1, 37]. Laut der WHO übersteigen die negativen Auswirkungen des Biodiversitätsverlustes auf die Gesundheit sogar die Risiken des Klimawandels [33], obwohl dieser deutlich stärker im öffentlichen Diskurs adressiert wird.

Ökologische Stabilität kann als Voraussetzung gesellschaftlicher und staatlicher Sicherheit verstanden werden [41]. Es ist essenziell, den Schutz der Biodiversität und ihrer Leistungen in nationale Sicherheitsstrategien zu integrieren, um langfristige Resilienz und Stabilität zu gewährleisten [14]. Als natürliche Infrastruktur tragen Ökosysteme auf vielfältige Weise zum Schutz und zur Versorgung der Bevölkerung bei, beispielsweise indem sie Trinkwasser bereitstellen, die Auswirkungen des Klimawandels abmildern oder die Grundlage für die Erzeugung von Lebensmitteln und Biomasse sind [14, 42].

Biodiversität sollte zukünftig intensiver im Zusammenhang mit Gesundheits- und Krankheitsfragen untersucht und im Rahmen der Wissenschaftskommunikation stärker in die Öffentlichkeit getragen werden [43]. Mittel- bis langfristig könnte sich der Biodiversitätsverlust ähnlich wie der Klimawandel als „Risikomultiplikator“ erweisen, der bestehende politische, soziale und wirtschaftliche Spannungen verschärfen kann [44]. Biodiversitätsschutz ist daher nicht allein eine ökologische, sondern auch eine sicherheitspolitische Aufgabe.

## Empfehlungen der Kommission Environmental Public Health

Die Kommission Environmental Public Health trifft vor diesem Hintergrund zusammenfassend folgende Feststellungen und gibt folgende konkrete Empfehlungen zur Sicherung und Fortführung der sektorenübergreifenden Zusammenarbeit zu Umwelt und Gesundheit:

1. **Umwelt und Gesundheit als strategisches Querschnittsthema institutionell verankern**
  Die Kommission Environmental Public Health empfiehlt, das Thema Umwelt und Gesundheit nachhaltig in den nachgeordneten Behörden des Bundesgesundheitsministeriums (Robert Koch-Institut [RKI] und Bundesinstitut für Öffentliche Gesundheit [BIÖG]) und des Bundesministeriums für Umwelt, Klimaschutz, Naturschutz und nukleare Sicherheit ([BMUKN], Umweltbundesamt [UBA], Bundesamt für Naturschutz [BfN] und Bundesamt für Strahlenschutz [BfS]) zu verankern. Hierfür wäre eine Forschungs- und Maßnahmen-Agenda hilfreich, die neben Umwelt- und Gesundheitsschutz auch Fragen der Gesundheitsförderung und Gerechtigkeit sowie Verhältnis- und Verhaltensprävention umfasst. Die enge Abstimmung zwischen dem Gesundheits- und dem Umweltsektor sowie weiterer Sektoren (Verkehr, Wirtschaft, Arbeit und Soziales u. a.) ist von zentraler Bedeutung für erfolgreiche und problemorientierte Analysen und darauf aufbauende politische und planerische Empfehlungen in diesem Bereich. Hier sind in den letzten Jahren vielversprechende Ansätze implementiert worden, die allerdings bei Weitem nicht ausreichen, um der Problemlage planetarer Krisen gerecht zu werden.
2. **Datenlücken schließen, um Handlungsmöglichkeiten von Gesundheitsfachkräften zu fördern**
  Entscheidend für eine enge Zusammenarbeit zwischen den Sektoren Umwelt und Gesundheit ist die Nutzung vorhandener Daten. Die beteiligten Einrichtungen sollten gemeinsam mit Forschungseinrichtungen die Nutzung und den Zugang zu behördlichen Daten abstimmen. Ziel ist es, einen Ansatz „Environmental Health in All Policies“ erfolgreich umzusetzen, der systematisch auch Fragen der Gerechtigkeit berücksichtigt („Environmental Health Equity in All Policies“) und konkrete Optionen für politische und planerische Maßnahmen aufzeigt. Für den Klimaschutz bedeutet das: Eine enge interdisziplinäre Zusammenarbeit ist notwendig – insbesondere zwischen Public Health, Klimafolgenforschung, Raum‑, Landschafts- und Stadtplanung sowie der Politik. Dabei müssen Aspekte sozialer Gerechtigkeit und Gesundheitsgerechtigkeit konsequent integriert werden [18]. Gesundheitsfachkräfte müssen in die Lage versetzt werden, klimabedingte Gesundheitsrisiken frühzeitig zu erkennen und als Treiber von Veränderungsprozessen „change agents“ angemessen darauf zu reagieren. Dafür erforderlich sind die Schulung und Weiterbildung von Gesundheitspersonal zum Umgang mit klimabedingten Krankheiten und die Entwicklung von Präventionsstrategien [1, 45].
3. **Bewusstsein für Gesundheitsrisiken stärken, Informationen bündeln und transparent offenlegen**
  Es sollten Kommunikations- und Edukationsformate angestoßen werden, mithilfe derer die Bürgerinnen und Bürger alle notwendigen Informationen zu umweltbezogenen Gesundheitsfragen, unabhängig von der Zuständigkeit, zentral auffinden. Das Bundesinstitut für Öffentliche Gesundheit (BIÖG) könnte hierfür eine geeignete Einrichtung sein. Die Kommission Environmental Public Health befürwortet eine transparente Kommunikation von Risiken und Ressourcen der Umwelt, die auch sozioökonomische Ungleichheiten und das Verursacherprinzip in Hinsicht auf Umwelt- und Gesundheitsbelastungen adressiert. Bezüge zwischen Umwelt und Gesundheit sollen transparent und im offenen Dialog mit der Bevölkerung diskutiert werden. Die entsprechenden Maßnahmen sollen fair und nachvollziehbar sein. Ein Beispiel ist die Mobilitätswende: Die Förderung umweltfreundlicherer Verkehrsmittel führt zur Verringerung der Luftverschmutzung sowie zu höherer körperlicher Aktivität und verbessert damit die Lebensqualität. Gesundheit und umweltbezogene Prävention müssen in den Mittelpunkt politischer Entscheidungen gerückt werden und es sollten transformative Maßnahmen ergriffen werden, die über bisherige Ansätze hinausgehen [1, 2, 46]. Soziale Innovationen sind dabei ebenso wichtig wie technologische Innovationen, um die gesundheitlichen Folgen des Klimawandels gerecht zu bewältigen und die nationale Sicherheit zu gewährleisten [1, 47]. Die Umsetzung von entsprechenden Maßnahmen, die zudem sogenannte Co-Benefits (d. h. positive Nebeneffekte) generieren, kann nicht nur die klimabedingten Gesundheitsrisiken reduzieren, sondern auch soziale, wirtschaftliche und sicherheitspolitische Vorteile mit sich bringen [1, 45, 48]. Entsprechend wichtig ist die politische Förderung der Erforschung und Umsetzung dieser Maßnahmen.
4. **Forschung zu Umwelt und Gesundheit stärken – bestehende Maßnahmen evaluieren**
  Für die zentralen Handlungsfelder Umweltschadstoffe, Klimawandel und in Teilen für Biodiversität liegt mittlerweile umfangreiches wissenschaftliches Wissen vor, sodass nunmehr die flächendeckende Umsetzung fortgesetzt oder begonnen werden sollte. Bestehende Forschungslücken müssen noch geschlossen werden.
  Die wissenschaftliche Befassung mit diesen Themen ist sicherheitsrelevant, weil eine Vernachlässigung von Umweltschutz und umweltassoziierten Gesundheitsfragen staatlich zu garantierende Lebensgrundlagen infrage stellt. Für die Kommission ist es zentral, dass die gesundheitlichen Folgen diverser Maßnahmen in diesen Handlungsfeldern gerade auch im Hinblick auf gesundheitliche Chancengerechtigkeit evaluiert und dokumentiert werden.
  Zukünftige Forschung zum Klimawandel sollte sich verstärkt mit den Auswirkungen des Klimawandels auf die menschliche Gesundheit, der Entwicklung von Frühwarnsystemen, der Identifizierung von Anpassungsstrategien für besonders gefährdete Gruppen und den Auswirkungen klimabedingter Migration auf die Gesundheitsinfrastruktur befassen [1, 46, 48]. Die Forschung zum Klimawandel sollte effizient organisiert und interdisziplinär koordiniert werden, um dem hohen Handlungsdruck gerecht zu werden [49]. Auch in der Nationalen Sicherheitsstrategie der Bundesregierung wird anerkannt, dass globale Umweltveränderungen (Klimawandel, Biodiversitätsverlust) die Lebensgrundlagen bedrohen und damit sicherheitsrelevante Risiken erzeugen – etwa durch Konflikte um Wasser oder Nahrung sowie durch Migration [50]. Im nationalen Sicherheitsrat findet ein integrierter Sicherheitsansatz allerdings bislang keine Berücksichtigung, das Umweltministerium ist nicht vertreten. Die Kommission Environmental Public Health kann hierbei begleitend tätig werden und ihren Sachverstand einbringen.

Zusammenfassend weist die Kommission Environmental Public Health darauf hin, dass es bei der Thematik Umwelt und Gesundheit um entscheidende zukunftsgerichtete, sicherheitsrelevante Fragen und Weichenstellungen geht, die für die Resilienz, Handlungsfähigkeit und langfristige Wohlstandssicherung Deutschlands von zentraler Bedeutung sind.

Die Kommission betont ausdrücklich, dass der enge Bezug von Umwelt und Gesundheit auch und gerade angesichts aktueller innen- und außenpolitischer Krisen und politischer Spannungen von zentraler Bedeutung ist. Umwelt- und Gesundheitsrisiken beeinflussen die gesellschaftliche Stabilität, die wirtschaftliche Leistungsfähigkeit und die Sicherheit Deutschlands unmittelbar. Deshalb müssen stringente Maßnahmen gerade jetzt geplant und umgesetzt werden.

Maßnahmen des Umwelt- und Klimaschutzes können helfen, Konflikte und Krisen zu vermeiden. In jedem Fall sollten militärische Auseinandersetzungen aus humanitären Gründen und Gründen des Umweltschutzes unbedingt vermieden werden. Sie tragen ihrerseits wesentlich zu den krisenhaften planetaren Veränderungen bei und gefährden so nicht nur akut, sondern auch dauerhaft lebenswerte und gesundheitsförderliche Lebensbedingungen weltweit. Aber selbst und gerade im Verteidigungsfall ist diese Thematik ebenfalls von zentraler Bedeutung für die Gesundheit der Bevölkerung sowie der militärischen und zivilen Einsatzkräfte, denn deren Resilienz und Einsatzfähigkeit hängt direkt von ihrer Gesundheit ab. Damit wird deutlich, dass Umwelt- und Gesundheitsschutz wesentliche Bestandteile einer modernen integrierten Sicherheitsvorsorge Deutschlands sind.

Auch vorhersehbare gesundheitliche Gefahren durch Umweltkontaminationen im Krisen- oder Verteidigungsfall erfordern eine starke Gesundheitsvorsorge und Überwachung der Umweltmedien. Sie kann nicht erst im Bedarfsfall aufgebaut werden und daher sollten bestehende Strukturen gestärkt und ausgebaut werden, u. a. um Kompetenz und Ausstattung für den Notfall vorzuhalten. Dies ist eine wichtige Investition in die Krisenvorsorge und Zukunftsfähigkeit Deutschlands und stärkt zugleich die Fähigkeit des Staates, auf gesundheitliche und umweltbezogene Gefährdungen wirksam zu reagieren. Die Vorbereitung auf Krisensituationen ist insofern auch zentral, um die Belastungen des bereits stark unter Druck stehenden Gesundheitssystems zu reduzieren.

Die Kommission steht in diesem Kontext zur wissenschaftlichen Diskussion und weiteren koordinierten Beratung gern zur Verfügung.

## Data Availability

Für das Positionspapier wurden keine Datensätze erstellt oder analysiert. Eine Angabe zur Datenverfügbarkeit ist deshalb nicht notwendig.
